# Design, characterization and experimental validation of a compact, flexible pulsed power architecture for ex vivo platelet activation

**DOI:** 10.1371/journal.pone.0181214

**Published:** 2017-07-26

**Authors:** Allen L. Garner, Antonio Caiafa, Yan Jiang, Steve Klopman, Christine Morton, Andrew S. Torres, Amanda M. Loveless, V. Bogdan Neculaes

**Affiliations:** 1 School of Nuclear Engineering, Purdue University, West Lafayette, Indiana, United States of America; 2 GE Global Research Center, Niskayuna, New York, United States of America; National Institutes of Health, National institute of Diabetes and Digestive and Kidney Diseases, UNITED STATES

## Abstract

Electric pulses can induce various changes in cell dynamics and properties depending upon pulse parameters; however, pulsed power generators for in vitro and ex vivo applications may have little to no flexibility in changing the pulse duration, rise- and fall-times, or pulse shape. We outline a compact pulsed power architecture that operates from hundreds of nanoseconds (with the potential for modification to tens of nanoseconds) to tens of microseconds by modifying a Marx topology via controlling switch sequences and voltages into each capacitor stage. We demonstrate that this device can deliver pulses to both low conductivity buffers, like standard pulsed power supplies used for electroporation, and higher conductivity solutions, such as blood and platelet rich plasma. We further test the effectiveness of this pulse generator for biomedical applications by successfully activating platelets ex vivo with 400 ns and 600 ns electric pulses. This novel bioelectrics platform may provide researchers with unprecedented flexibility to explore a wide range of pulse parameters that may induce phenomena ranging from intracellular to plasma membrane manipulation.

## Introduction

### Biological effects of electric pulses

Depending upon their intensity, duration, rise- and fall-times, and repetition rates, pulsed electric fields (PEFs) may induce multiple effects on biological cells, including temporary permeabilization of the plasma membrane to facilitate molecular delivery [1], complete rupture of the plasma membrane for liquid sterilization [2], irreversible electroporation for cancer therapy [3,4], permeabilization of tumors to facilitate drug delivery in electrochemotherapy [4,5], direct induction of apoptosis for cancer therapy [6], formation of small nanopores to facilitate ionic transport into neural cells [7] or platelets [8], and the permeabilization of the membranes of intracellular structures, such as the mitochondria [9] or intracellular calcium stores [10]. One can generally consider the impact of pulse duration as a continuum, with submicrosecond pulses tending to focus their effects on intracellular structures while microsecond to millisecond pulses generally induce plasma membrane effects [11]. It is important to note that while submicrosecond pulses still induce plasma membrane effects, such as plasma membrane permeabilization [12–13], the membrane effects are less intense than those induced by microsecond and millisecond pulses. Similarly, microsecond and millisecond pulses may still induce intracellular effects [14], but the preponderance of the effects will occur at the membrane level.

One may consider a biological cell electrically as a combination of resistors and capacitors with PEF-induced effects arising due to membrane charging [15]. Because of their smaller size, the intracellular organelles will be fully charged by shorter PEFs before the full cell; therefore, one would anticipate shorter PEFs to target intracellular structures prior to the plasma membrane [15]. Electric models solving the Laplace equation show that shorter duration PEFs will induce membrane voltages typical for electroporation (on the order of hundreds of millivolts to one volt [16]) for intracellular organelles, while longer pulses will induce these voltages on the plasma membrane. From a frequency perspective, AC fields containing higher frequency components (e.g. above MHz) will more likely impact intracellular structures, while lower frequency components (e.g. kHz) will more likely influence the plasma membrane [17–18]. One can similarly consider PEFs in the frequency domain by taking the Fourier Transform and showing that pulse duration and rise- and fall-times will impact the frequency content [19] and, therefore, impact the specific biological effects of the fields [17]. Short pulses with fast rise-times will more likely impact intracellular structures than those with slower rise-times and longer durations [15,20], although these shorter pulses will still induce plasma membrane effects [12–13].

While these effects have been the basis for ongoing studies over the years, it is generally challenging to design a flexible pulsed power system that provides full control over rise-time, repetition rate, and pulse duration for tunability in inducing specific biological effects. Additionally, it is advantageous to design a single pulse generator capable of delivering pulses to loads of various load resistance without requiring external matching resistors, as is common among many pulse generators designed for a single impedance [13, 19, 21]. This would open opportunities to use a single device to electrically stimulate cells in both low conductivity (e.g. typical electroporation buffers) and higher conductivity solutions (e.g. cell growth media, blood, platelet rich plasma).

### Overview of pulse generators for bioelectrics research

Pulse generators, distinguished by their unique ability to provide high intensity, short duration electric pulses, have been designed, developed, and used in industry [22–23], military [24], medicine [25], environmental remediation [23,25], and agriculture [26] over the past several decades. Depending upon the circuit topologies and switching devices, pulse generators vary widely in performance, such as output voltage, rise/fall time, pulse width, repetition rate and load dependency; therefore, one must carefully design pulse generators according to the load requirements for the specific application. Marx generators and pulse forming lines are the two most relevant pulse generator topologies in the context of our work. Marx generators [27] are commonly used for high voltage applications (>100 kV). A Marx generator is basically a capacitive adder, where *N* capacitors are charged to *V*_*C*_ in parallel. Closing all the switches connects the capacitors in series, which applies *NV*_*C*_ to the load. Early pulse generators often relied on Marx generators and spark gap switches, which are usually bulky and require a relatively long recovery time, which limits the repetition rate. Solid-state switch based Marx generators, such as the cascaded boost converter [28], can achieve fast rise times and flat-top shapes with high repetition rates, controllable output voltages and pulse widths, and much longer lifetimes.

Pulse forming lines (PFLs) [29] or Blumlein transmission lines [19, 30] are by far the most common topology used for short pulse generation, especially for generating square pulse shapes. PFLs can deliver exact energy storage with specified pulse shape, but require matching the load impedance to the PFL characteristic impedance. The pulse width is determined by the physical length of the transmission line structure and its material properties; therefore, it lacks the flexibility to easily vary the pulse width. PFLs can usually provide very high repetition rates.

One of the challenges for biomedical PEF applications is designing a single pulse generator capable of delivering pulses with durations ranging from nanoseconds to microseconds and beyond while maintaining compactness for versatile bioelectrics research or clinical applications, such as platelet activation [8], and achieving sufficiently high voltages (~10 kV). Multiple groups have developed concepts enabling multiple pulse durations and pulse patterns by controlling switching cycles, but many of the current approaches have drawbacks for the specific applications desired here or cannot be readily adapted to enable tunability over the required range of pulse durations.

One example involved incorporating a metal-oxide-semiconductor field–effect transistor (MOSFET) as a switch on each end of the Blumlein line to generate a 1 kV pulse across a 100 Ω load with durations between 8 and 60 ns [31]. This approach was useful for applying pulses across closely spaced electrodes under a microscope, but will not provide the necessary voltage flexibility for applying electric fields to a cuvette. Additionally, it does not provide pulse duration flexibility from nanoseconds to tens of microseconds that would enable a complete parametric study of bioelectrics effects on cells using a single instrument, such as exploring the transition from intracellular to plasma membrane dominated effects. Another study further modified a Blumlein system to provide flexible pulse parameter delivery to either a microscope slide or a cuvette [32]. Based upon coaxial transmission lines, this revised Blumlein system could apply 30–200 ns pulses up to 1 kV to cell suspensions on microscope slides, but again does not provide the capability of longer duration pulses [32]. Another modification of the Blumlein configuration enabled pulse repetition rates up to 1.1 MHz with pulses of variable duration and polarity, but was again limited to submicrosecond duration [33]. Realizing the Blumlein architecture with a microstrip line configuration containing interchangeable lines enabled matching electric pulses to multiple loads with variable pulse duration, amplitude, repetition rate, and polarity in cuvettes and under microscope slides; however, the pulse durations remained submicrosecond [34].

Higher voltages can be obtained by using a solid-state linear transformer driver (LTD) stack [35]. In one example, an LTD stack of thirty modules consisting of twenty-four power (MOSFETs) as switches, generated up to ~29 kV with a maximum current of ~240 A and variable pulse width of 50–170 ns [35]. LTDs could be a useful alternative, but this particular design was limited for our application by the narrow range of pulse duration and relatively large size (~23 kg). Additionally, the LTD topology approach is limited in pulse width flexibility because the PEF is transmitted through a magnetic structure, which can saturate for longer pulse durations. A subnanosecond pulse generator capable of delivering 250 kV with variable pulse duration into a high impedance load was also developed, but would not be easily scalable to microsecond duration [36]. Another study used photoconductive semiconductor switches to generate pulses of various shapes with maximum amplitudes of 6.9 kV for nanosecond and subnanosecond pulses [37]. One can also utilize telescopic antenna and ferrites in the pulse-forming line of the generator to produce 1–10 ns pulses with tens of kilovolts of amplitude and subnanosecond rise-times [38]. Another approach used two avalanche transistor stacks as switches to generate 800 V to 3.8 kV pulses with 5–38 ns pulse widths [39]. Alternatively, one can configure two MOSFET based 10 kV switches in differential mode to generate 1 kV to 10 kV pulses with rise-times shorter than 5 ns [40]. Another approach entailed designing a voltage compressor circuit using transmission lines, although the length of the lines (6 m) makes this approach impractical for applications requiring compactness [41].

Marx banks have been modified to provide flexible pulse durations [42–43]. For instance, a circuit employing two parallel Marx generators utilizing bipolar junction transistors (BJTs) as closing switches generated 1–10 kV pulses with pulse widths and pulse rise- and fall-times on the order of nanoseconds to tens of nanoseconds for generating microplasmas; however, this approach was not extended to longer pulse durations [42]. Another study modified the Marx structure by using a solid-state boosting front-end and H-bridge output stage to enable modification of pulse duration and polarity for microsecond pulses [43]. Another approach generated 100–3000 V, 100–200 μs pulses by modifying the biasing supply of the high voltage MOSFET based switch [44].

Often, the approaches used for creating adjustable submicrosecond pulses are not easily adapted to generating longer pulses [44] and vice versa. Thus, this paper focuses on developing a novel modification of a Marx bank architecture for flexible selection of pulse parameters from nanoseconds to microseconds while also being able to apply electric pulses to low (buffer solution) and high (blood, platelet rich plasma—PRP) conductivity solutions without requiring an external matching resistor. We will then use the new device to assess the impact of pulse duration on platelet activation [8, 45]. We choose this application because although bovine thrombin, the current state of the art method of activating platelets, is effective, it can have adverse side effects for the patient. For instance, patients develop cross-reacting antibodies in 30% of the treatments [46] with the average costs of thrombin-associated immune-mediated coagulopathy ranging from $16,584 to $163,072 per patient [47]. This compact pulsed power platform could be the foundation for a new class of bioelectrics research instruments for clinical use or laboratory use for exploring effects ranging from intracellular to plasma membrane manipulation.

We note that our proposed design differs from that of Ref. [35] by enabling the extension of pulse durations beyond 500 ns, which is a limitation because of the resonant equivalent circuit [48] and the properties of the magnetic material. Our design can provide pulses from tens of nanoseconds to beyond tens of microseconds by combining capacitive energy storage and continuous power transmission. Capacitive energy storage provides energy for short pulses, while the capacitances are simultaneously connected to power supplies to provide continuous power to the load without saturating the magnetic structure. The topology also enables pulse sharpening either by using a second breakdown mode in the semiconductor device (e.g. a thyristor) or by using a secondary magnetic structure (as a magnetic switch).

The Materials and Methods section summarizes the design and preliminary load testing of the tunable pulsed power device. We provide details on benchmarking the pulsed power system to buffer solutions of various conductivities and present results for applying electric pulses for platelet activation in the Results section. We summarize the implications of the design and results in the Discussion.

## Materials and methods

### Load definition and impedance measurement

Load impedance is a critical parameter for pulse generator design. In addition to designing a pulse generator to provide flexibility in pulse duration (from tens of nanoseconds to tens of microseconds), pulse shape, pulse amplitude, and number of pulses, this pulse generator may also treat both low conductivity and high conductivity samples, such as blood and PRP. For this application, we note that blood and PRP have similar electric behavior [45]. Successful demonstration of ex vivo platelet activation with this pulse generator could motivate its development as a clinical grade instrument in future embodiments. The need to successfully treat high conductivity biological samples in typical electroporation cuvettes prompts the choice of components with high current capability for our design. Once the pulse generator can treat high conductivity samples (e.g. the electronic components have high current capability), the same pulsed power topology could also electrically stimulate lower conductivity samples, such as typical electroporation buffers.

We performed impedance measurements to electrically characterize 2 mm cuvettes with high conductivity loads: phosphate-buffered saline (PBS) and human whole blood. We do not measure PRP impedance since whole blood and PRP have similar electrical behaviors [45]. We then used the electrical measurements of PBS and whole blood cuvettes to design, simulate and optimize the performance of our intended pulse generator topology while also identifying the electronic components to be utilized in building the prototype

We used an Agilent 4294a impedance analyzer to electrically characterize three PBS (Phosphate-Buffered Saline; 1X PBS pH 7.4 Gibco Cat # 10010) and two human whole blood samples in standard electroporation cuvettes with a 2 mm electrode gap over a wide range of frequencies. The cuvette’s geometry approximates a parallel plate capacitor with an empty cuvette essentially a capacitor of capacitance *C* = ε*A*/*d*, where ε is the permittivity of the material of the dielectric between the plates, *A* is the cross-sectional area of the electrode, and *d* is the separation distance between the two electrodes. Using *A* = 2.15cm^2^ (9.7 mm × 22.21 mm), *d* = 2 mm, and air as the dielectric for an empty cuvette (ε = ε_0_ = 8.854×10^−12^ F/m) gives a capacitance of 9.52×10^−13^ F. This shows that the parasitic capacitance introduced by the cuvette has a very limited, if any, effect on sample impedance up to tens of MHz.

For the PBS measurements, we filled the cuvette with different quantities of PBS to vary the level above the upper edge of the electrode, as summarized in Table 1. These measurements permitted evaluation of the effects of overfilling the cuvettes with biological samples, which could happen if the operator adds sample in excess of the nominal volume. Thus, these studies would demonstrate the impact of various cuvette fill levels on the sample impedance and the implications on pulsed power instrument performance, specifically the features of the output pulse. For instance, treating a load with a higher capacitance than that for which the pulsed power device is designed would increase the rise-time of the electric pulse.

**Table 1:** Equivalent resistance and capacitance of three PBS samples representing different levels of cuvette overfilling.

	Height above edge (mm)	Capacitance (nF)	Resistance (Ω)
PBS1	5	520	6.7
PBS2	11	650	6.6
PBS3	16	750	6.7

**Fig 1:**
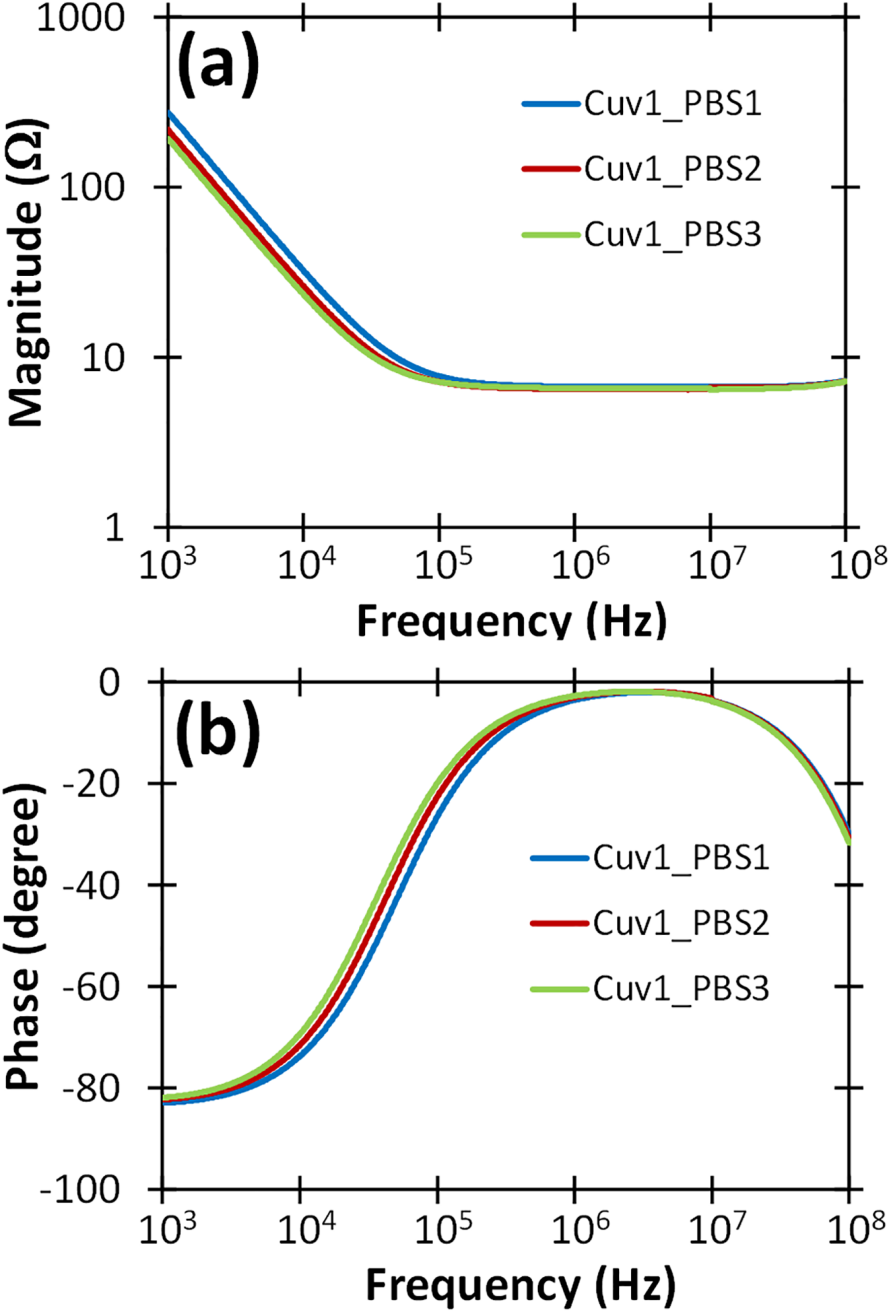
Measured (a) magnitude and (b) phase of the impedance of three PBS samples in cuvettes in the frequency domain using an impedance analyzer with a 500 mV sine wave with a variable frequency from 1 kHz to 100 MHz.

Fig 1 shows the measured impedance of the PBS samples for each cuvette fill level with an impedance analyzer set to deliver a 500 mV sine wave with a variable frequency from 1 kHz to 100 MHz. The measurements demonstrated that the system/sample is equivalent to a capacitor in series with a resistor up to tens of MHz frequency range. The capacitor’s impedance decreases linearly with frequency on a log-log scale while the resistor’s impedance remains constant with increasing frequency; therefore, the load impedance is high at low frequency and decreases until the resistor’s contribution is dominant. The equivalent impedance (capacitance and resistance obtained by the impedance analyzer to best represent the electrical behavior over the measured frequency range) for the three PBS levels, summarized in Table 1, show that the PBS level noticeably changes the capacitance while minimally impacting the equivalent resistance. Electrically, overfilling the cuvette increases the load capacitance, which may increase the rise-time while not significantly altering the peak voltage and current. These initial measurements allowed us to assess the impact of slight variations in cuvette filling on the resulting impedance to elucidate the potential ability of our pulse generator to still provide matched signals for slight variations in the biological load. The capacitance associated with the cuvette’s geometry, which is in parallel with the sample’s equivalent impedance, has negligible effect in the measured range due to its extremely small value.

**Fig 2:**
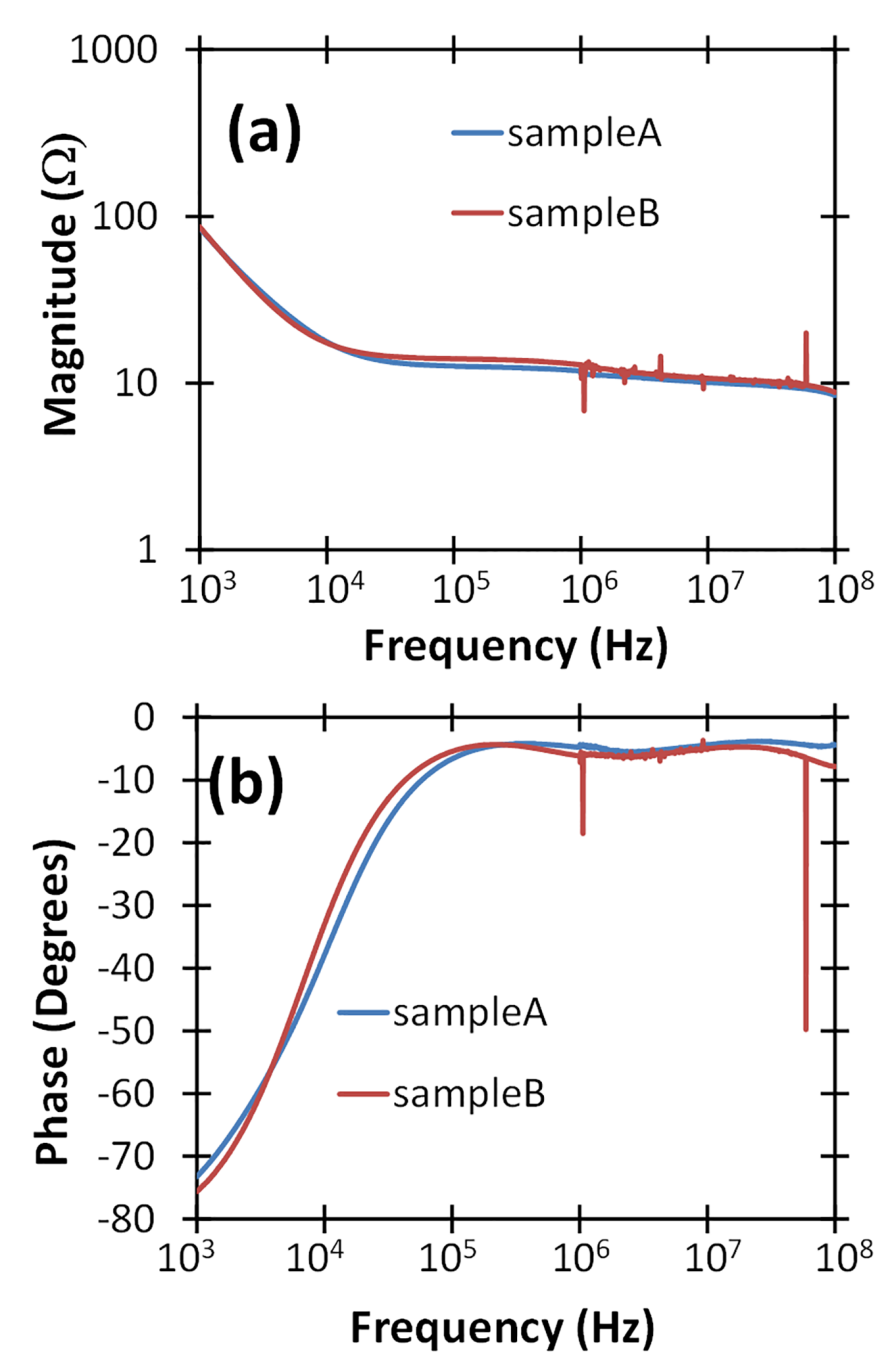
Measured (a) magnitude and (b) phase of the impedance of two whole blood samples in 2 mm cuvettes in the frequency domain, using an impedance analyzer with a 500 mV sine wave with a variable frequency from 1 kHz to 100 MHz.

Fig 2 shows the impedance of two fresh human blood samples in 2 mm cuvettes filled to the top edge of the electrode (optimum cuvette filling). Like PBS, the impedance of human blood is equivalent to a capacitor in series with a resistor; however, human blood is more capacitive and less conductive than PBS with a capacitance of approximately 1.5 μF and a resistance of approximately 10 Ω, as measured in these tests. Even for blood samples, the parallel parasitic capacitance of the cuvettes plays a negligible role in sizing the pulse generator. Our pulse generator design targeted optimum pulse rise time for the more challenging load–whole blood, with much higher capacitance.

**Fig 3:**
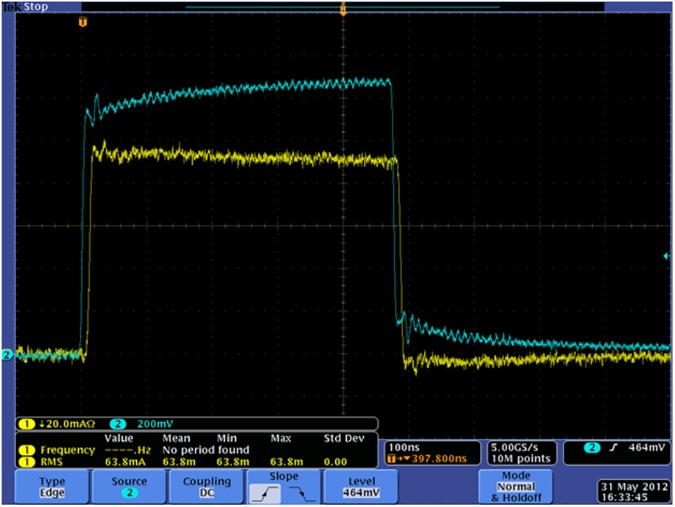
Low voltage test using a function generator to determine the resistance of blood sample A (voltage (blue): 200mV/div, current (yellow): 20mA/div. time scale: 100ns/div).

We also measured voltage and current across blood samples in 2 mm cuvettes exposed to low voltage pulses from a function generator. Fig 3 shows the measured voltage and current waveform following the treatment of sample A with a 500 ns pulse (this is just one example; the trends for sample B are similar–data not shown here). The voltage and current are approximately 1200 mV and 100 mA, respectively, which yields a 12 Ω resistance for the tested sample. Fig 3 shows that the current waveform closely follows the voltage waveform, which indicates the resistance dominates the load impedance and dictates the peak current for a given voltage profile. For a typical voltage pulse intended for platelet activation, the peak voltage is approximately a few kilovolts [8], meaning that the peak current will be on the order of a few hundred Amperes. This imposes significant design challenges since the pulse generator must simultaneously provide very high voltage and high current. These challenges require an advanced circuit topology and an appropriate selection of the semiconductor devices.

Thus, we have determined the electrical behavior of both PBS and whole blood as a function of frequency to guide the development of the pulse generator for platelet experiments, while also demonstrating that overfilling a cuvette has negligible impact on resistance and requires a large degree of overfilling to impact the capacitance.

### Pulse generator design

Upon characterizing the intended load, we next undertook the design process and component selection. The proposed pulse generating system is derived from a Marx generator topology and has been designed to maximize the ability to control key pulse parameters, such as amplitude, duration and pulse shape, while efficiently treating high conductivity biological samples. Typical Marx approaches charge the capacitors in parallel at low voltage and, through appropriate switching, are connected and discharged in series into the load. In most traditional topologies, the discharge is uncontrolled and concludes when the energy stored in the capacitors is delivered into the load. While our proposed pulse generator relies on a multistage approach, it differs from a Marx approach in four main ways.

First, as proposed elsewhere [49], we replaced the spark gaps and isolating inductors with controllable switches to enhance pulse generator control. This makes the pulse generator voltage independent of the spark gap triggering voltage and allows it to be turned on upon charging the capacitors to the optimal voltage, corresponding to the most efficient transfer of voltage or energy to the load. In this case, optimal voltage refers to the voltage across each stage, so one sets the voltage across each stage to an optimal value. The user can then input the optimal value to enable 1) the application of different voltage increments tailored to the load and 2) reduced ripple for high current loads by appropriately engaging and disengaging different stages. The buffer capacitors are charged through a resonant converter at 32 kHz. The refresh rate on the capacitors corresponds to that frequency, which can be adjusted by software to match diverse loads.

Using controllable switches also makes turning off the pulse independent of the load current, unlike spark gaps, which turn off when the load current reaches zero. This last capability is critical for applying square waveform to the load, even when the load absorbs energy from the pulse generator.

Also, as opposed to traditional method of charging the capacitors in parallel with a single voltage source, our proposed approach charges each capacitor independently with a dedicated power supply. Moreover, the capacitors are always connected to the charging power supplies, which allows for an “extended” pulse length even when the load absorbs considerable energy. The voltage sources can be designed to compensate for the “signature” droop of Marx generators for a given load and maximum pulse length, reducing the effective droop to a small percentage of the applied voltage (the acceptable droop, if any, would be a design input parameter).

Additionally, contrary to a traditional Marx generator and adding to the device’s flexibility, our proposed approach allows the connection of only a subset of the capacitors to the load. Thus, capacitive stages can be switched in and out when the voltage requested is just a fraction of the pulse generator’s full capability. For instance, a set of capacitances being discharged into the load can be disconnected while, simultaneously, a second set of capacitances fully charged can be switched in. The disconnected capacitor(s) can recharge at a much faster rate. This operation exposes the load to a smaller ripple compared to the traditional approach with minimal reduction in voltage applied to the load. Finally, our design for localized pulse sharpening (i.e. reduction of pulse rise-time) leverages the secondary breakdown of solid-state thyristors to sharpen the pulse “per stage” rather than only globally, which enhances operational flexibility. For instance, if the load requires pulse superposition or composite pulses, localized pulse sharpening will allow the sharpening of every step of the overall pulse

**Fig 4:**
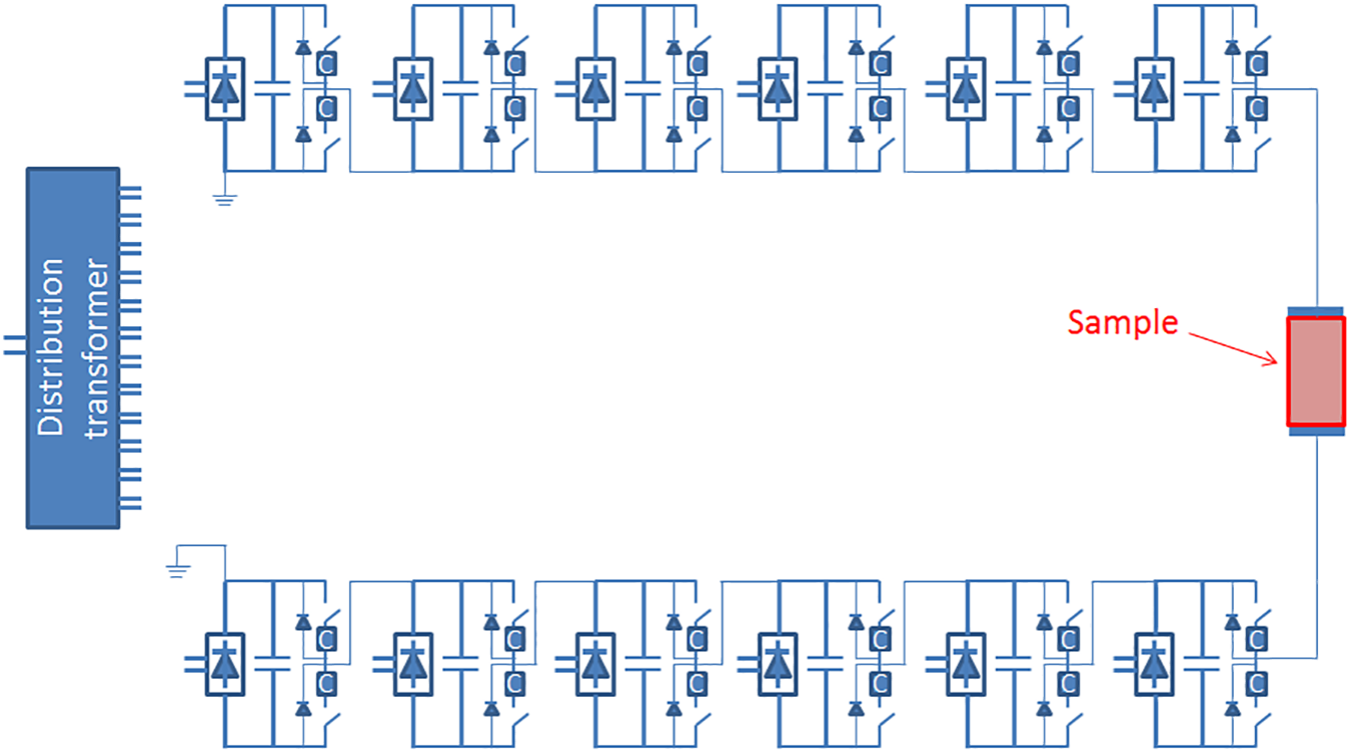
Circuit diagram of the twelve stage pulse generator architecture initially designed and simulated.

Typical of Marx-like structures, this pulse generator is designed as a modular structure. We initially designed and simulated a twelve stage pulse generator (c.f. Fig 4), while constructing and testing a six stage pulse generator as our first prototype. Future embodiments of this platform can easily extend the number of stages from six to twelve; however, the platelet activation experiments required only a few kilovolts, so six stages sufficed for this application. The initially simulated pulse generator includes six stages to produce a positive pulse, while the other six stages produce a negative pulse, creating a maximum voltage on the load equal to twelve stages (i.e., if each stage produces 1 kV then the pulse generator can produce 6 kV positive and 6 kV negative for an overall voltage of 12 kV on the load). We point out that even if a portion of the circuit produces negative voltage to ground, the load will only be exposed to positive voltage. Even if the simulated pulse generator includes twelve total stages, and the constructed prototype includes six stages, this topology is suitable for different numbers of stages. The use of stages to create both positive and negative polarities reduces the instrument’s footprint because the dielectric insulation thickness increases more than linearly with voltage. Thus, operating between +6 kV and -6 kV will make the device much more compact than operating at 12 kV.

**Fig 5:**
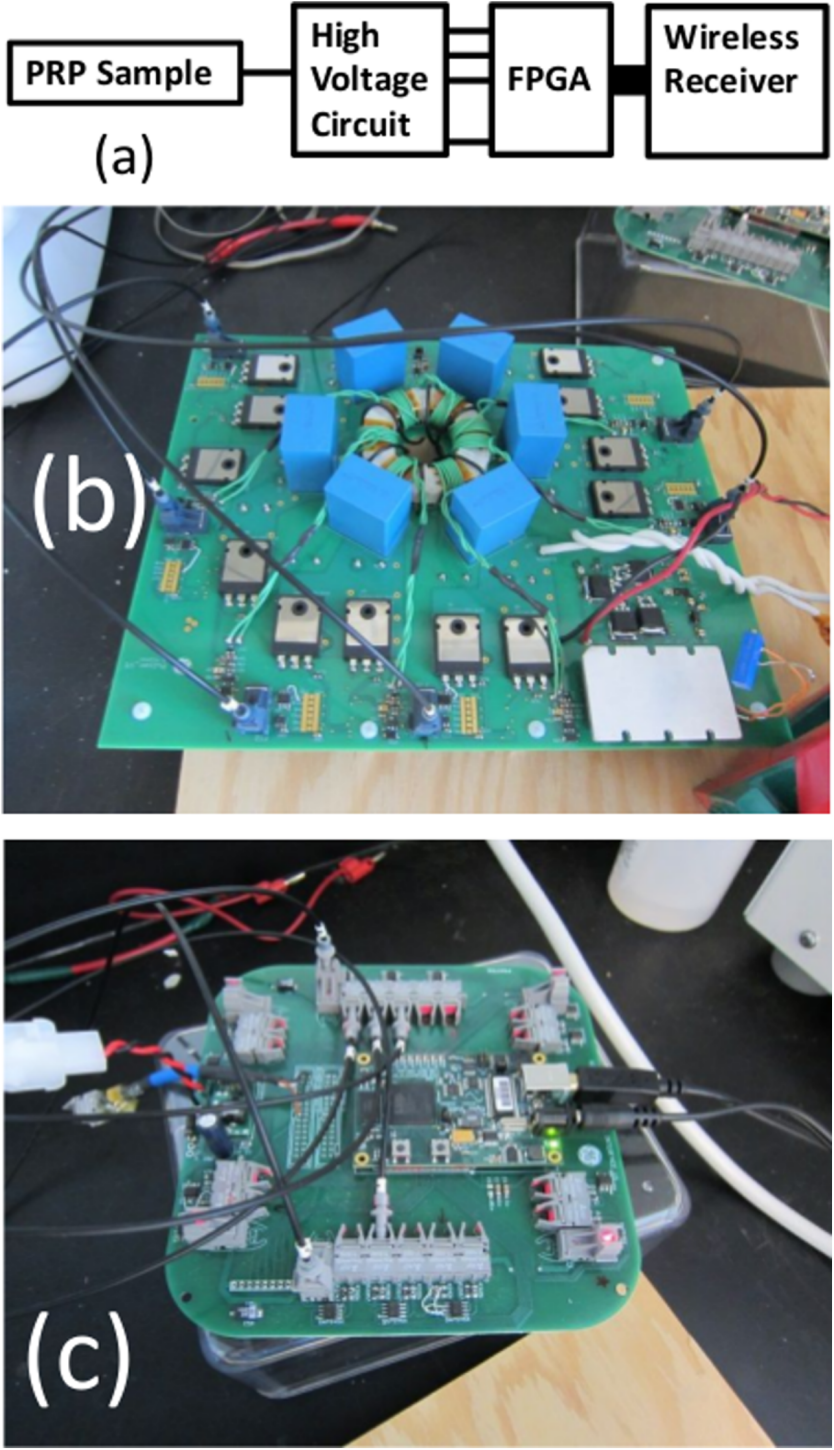
(a) Pulsed power system block diagram and key components, including the platelet rich plasma (PRP) within an electroporation cuvette as the load. Photographs of the (b) high voltage circuit board and (c) control board.

Fig 4 shows the overall circuit schematic of the device in the twelve stage embodiment. Fig 5 shows a block diagram of the constructed six stage pulsed power system and photographs of key system components within the setup. As mentioned previously, the constructed prototype consists of six stages, each of which includes a set of 5 μF B32774D1505K film capacitors (one on top and one on the bottom) with as low of equivalent series resistance and equivalent series inductance as possible, a controllable voltage source, a set of switches consisting of two IGBT transistors (IXYK 120N120C3) per stage with a peak voltage of 1.2 kV and a peak current of 660 A for 1 ms, and a transformer with a magnetic core made of nanomagnetic material. The pulse generator is controlled remotely by a computer that interfaces with the hardware via a Field Programmable Gate Array (FPGA). This allows remote placement of the control station to ensure operator safety when needed. The FPGA and the computer in the control station can be connected wirelessly (c.f. Fig 5) or through a USB connector. Once the operator selects the desired pulse sequence and pulse features (such as duration and amplitude) through a dedicated graphical user interface (GUI), the FPGA receives the information and, using a dedicated algorithm, controls the single stages to produce the desired pulse shape and sequence of pulses if applying more than one pulse. Using fiber optics to connect the FPGA with the high voltage elements and locally powered gate drive circuitries assures galvanic isolation between the FPGA and the high voltage side. The system utilizes an AC/DC and a DC/DC power supply. The AC/DC (LS100-24) power supply connects to the main power (120 Vac) and provides 240 Vdc to power the cooling fans. The DC/DC (Vicor V24C36C100BV) provides 36 Vdc to a switching H-bridge, which is then connected to a resonance tank and, finally, to the primary of the main transformer. The H-bridge is operated in hyper-resonant mode.

In addition to controlling the activation, deactivation, and duration of each stage, these systems also control the charging voltage of the capacitor banks, which further increases this instrument’s flexibility.

### Platelet rich plasma experimental setup

#### Platelet rich plasma (PRP) preparation

We handled whole blood and samples derived from whole blood, such as platelet poor plasma (PPP) and PRP, using universal precautions. Single units of human whole blood (WB) from individual donors were purchased and shipped overnight at room temperature from a commercial vendor (Bioreclamation, Westbury, NY). All studies used acid citrate dextrose (ACD) as an anticoagulant. We next report pilot experimental results for blood from one donor.

For a single preparation of PRP, 60 mL of whole blood (WB) was drawn from the original unit of blood received from Bioreclamation using syringe and needle components from the SmartPReP2 APC+ PRP preparation kit (Harvest Technologies, Belton, TX). The whole blood was injected into the separation component and centrifuged using the Harvest Technologies centrifuge. Complete separation using centrifugation (approximately 2300–2500 RPM, according to the manufacturer of the centrifuge) typically takes fifteen minutes. We used forces of 1250 g and 1050 g for the first and second spins, respectively. We then collected PRP with a yield of ~10mL per 60 mL of WB used. PRP was maintained at room temperature for all experiments. The enrichment of platelets in PRP was three-fold higher than in the original WB sample.

All biochemical reagents were prepared and stored on ice on the day of experiment. Bovine thrombin (BioPharm Laboratories, Bluffdale, UT, catalog #91–010) was prepared in saline solution (0.9% NaCl) at a 10 units/μL stock concentration to allow for 1:10 (vol/vol) standard dilution in all experiments. Final concentration after addition to PRP was 1 U/μL. CaCl_2_ (Sigma Aldrich, St. Louis, MO) was prepared at 1 M stock concentration to allow for 1:100 (vol/vol) standard dilution in all experiments. The final concentration after addition to PRP was 10 mM CaCl_2_, which is a typical concentration used in electric pulse platelet activation [50]. Platelet activation is done within 10 minutes from PRP separation from the whole blood.

#### Thrombin-mediated activation of platelet rich plasma

Bovine thrombin (stock concentration 100U/μL) was added to 0.5 mL of PRP with 5 μL stock CaCl_2_ in 2 mm cuvettes (Molecular BioProducts/Thermo Scientific, Pittsburgh, PA, catalog #21-237-2) at 1:100 dilution (final concentration 1U/μL) and then incubated at room temperature. Clotting with bovine thrombin typically occurred within approximately thirty seconds. Activated platelets were removed from the cuvette and then centrifuged at 10,000 rpm for ten minutes in a 1.5mL Eppendorf tube. The resulting supernatant was pipetted from the tube and either used immediately or stored at or below -20°C.

#### PEF-mediated activation of platelet rich plasma

For each experiment, 5 μL stock CaCl_2_ was added to 0.5 mL of freshly prepared PRP in a 2 mm cuvette (Molecular BioProducts catalog #21-237-2); the sample was subsequently exposed to one electric pulse and incubated at room temperature for approximately fifteen minutes (clotting occurs within approximately 5 minutes).

A Tektronix DPO4104 oscilloscope and a Tektronix P6015A high voltage probe were used to measure the voltage pulses applied to cuvettes with PRP for activation. Electrical current was measured using a Pearson probe, model 110.

The electrically activated platelets were removed from the cuvette and centrifuged at 10,000 rpm for ten minutes in an Eppendorf tube. The supernatant was pipetted from the tube immediately after the pulse and either used immediately or stored at or below -20°C.

#### Growth factor measurements

We measured the release growth factor using the following two commercial kits: Human/Mouse PDGF-AA Immunoassay (R&D Systems, Minneapolis, MN, Catalog # DAA00B) and Human EGF Immunoassay (R&D Systems, Minneapolis, MN, Catalog #DEG00, SEG00, PDEG00). PDGF-AA (platelet derived growth factor) and EGF (endothelial growth factor) required supernatant dilutions of 1:10 and 1:20, respectively. We directly followed the manufacturer’s protocol for each assay without deviation.

## Results

### Initial pulse generator validation

**Fig 6:**
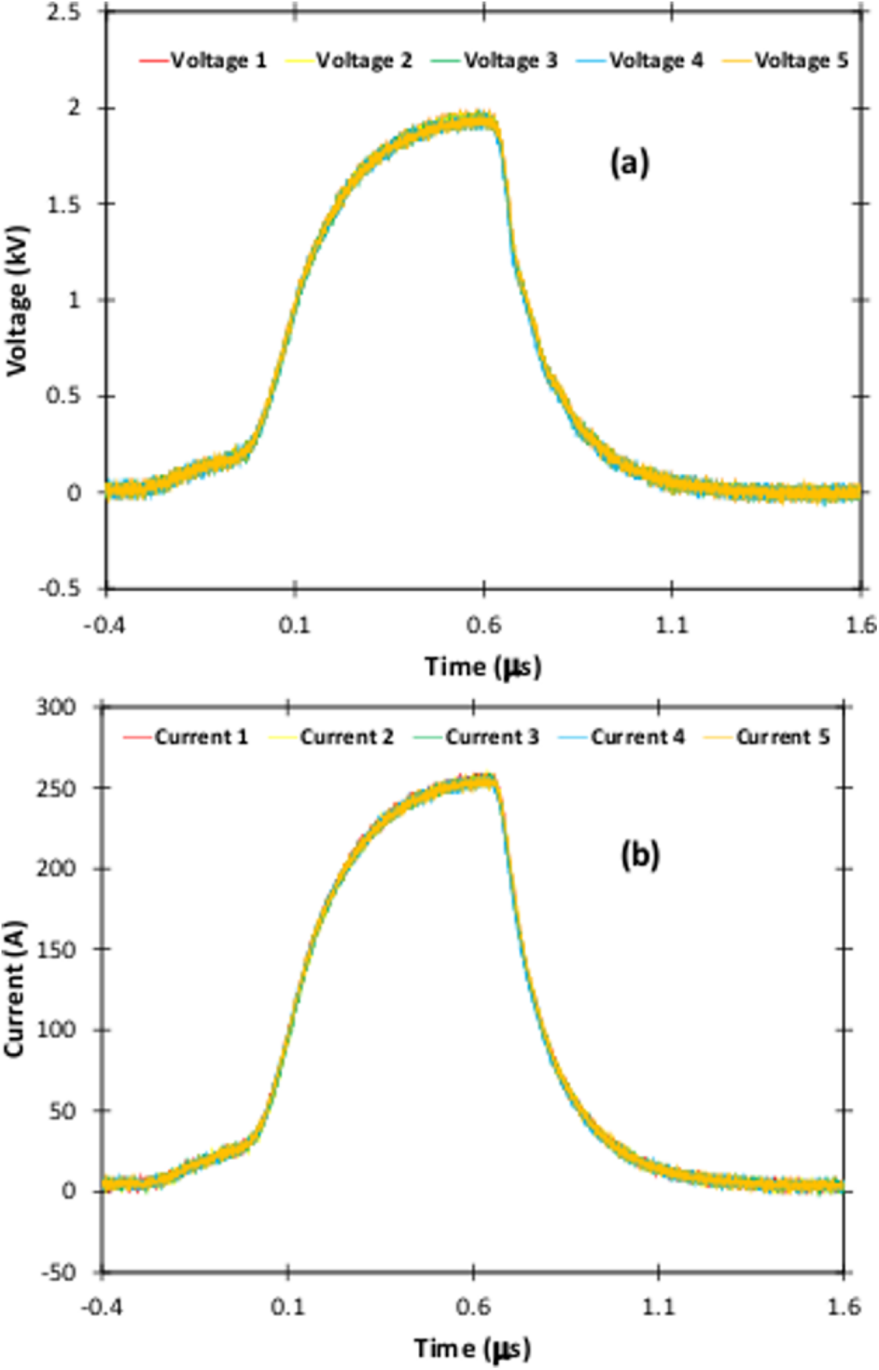
Measured (a) voltage and (b) current waveforms following application of five electric pulses to a PBS sample with one minute between pulses to demonstrate the pulse generator’s excellent reproducibility.

**Fig 7:**
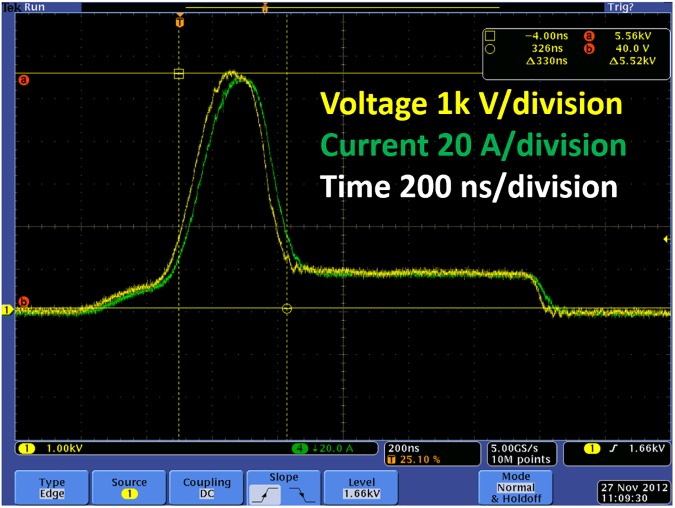
Example of pulse shaping with the GE pulse generator showing a composite pulse with 5.5 kV and 1 kV peak voltages, 330 ns and 700 ns pulse durations, and a peak current absorption of approximately 110 A across a resistor.

**Fig 8:**
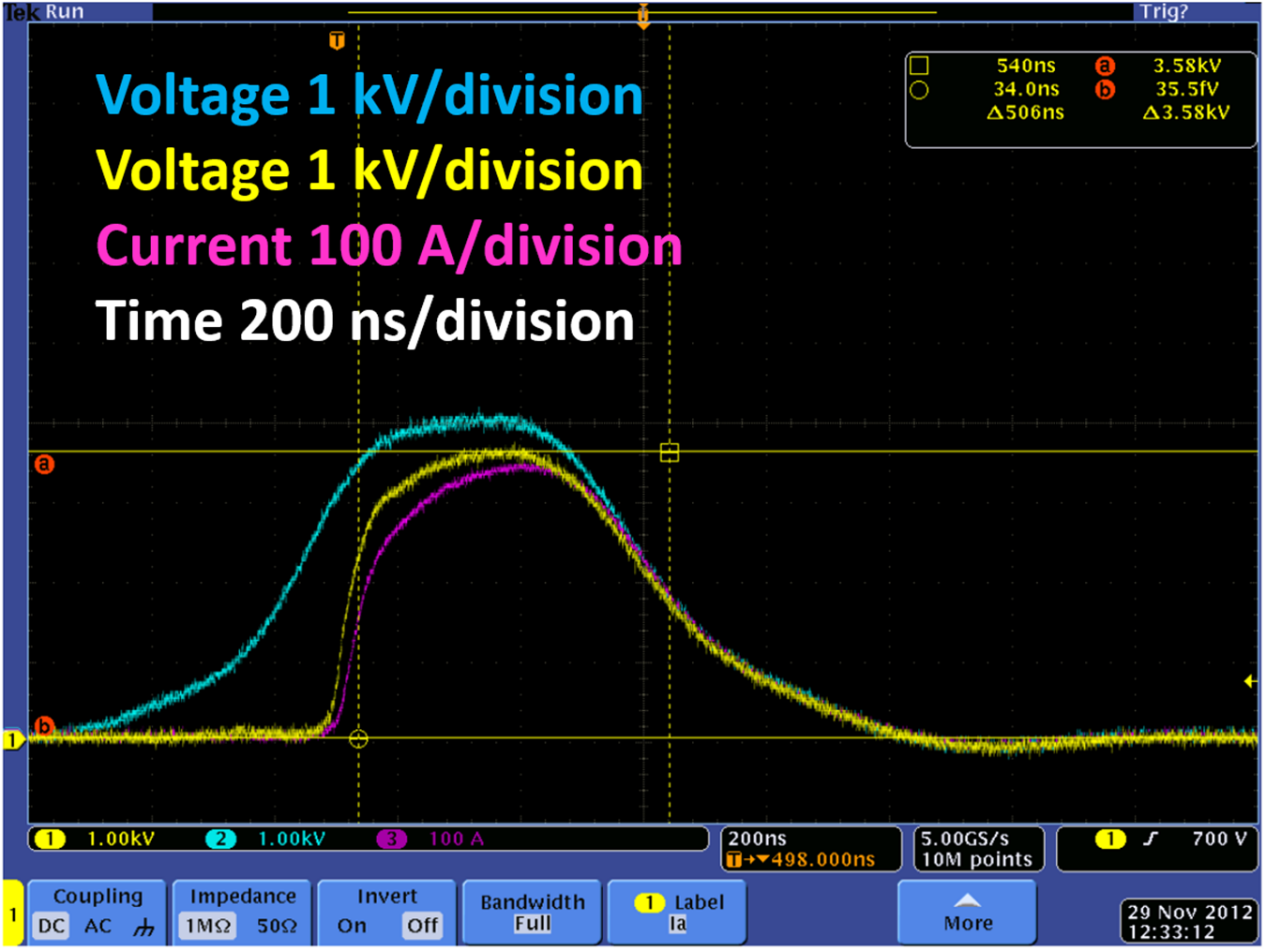
Solid state pulse sharpening–yellow trace, vs. voltage pulse without pulse sharpening–blue trace. The pink trace is the current waveform for a sharpened pulse (whole blood in a 2 mm cuvette was used as a load). The pulse duration of the sharpened pulse is 500 ns with a peak voltage of 3.58 kV and peak current of 358 A.

**Fig 9:**
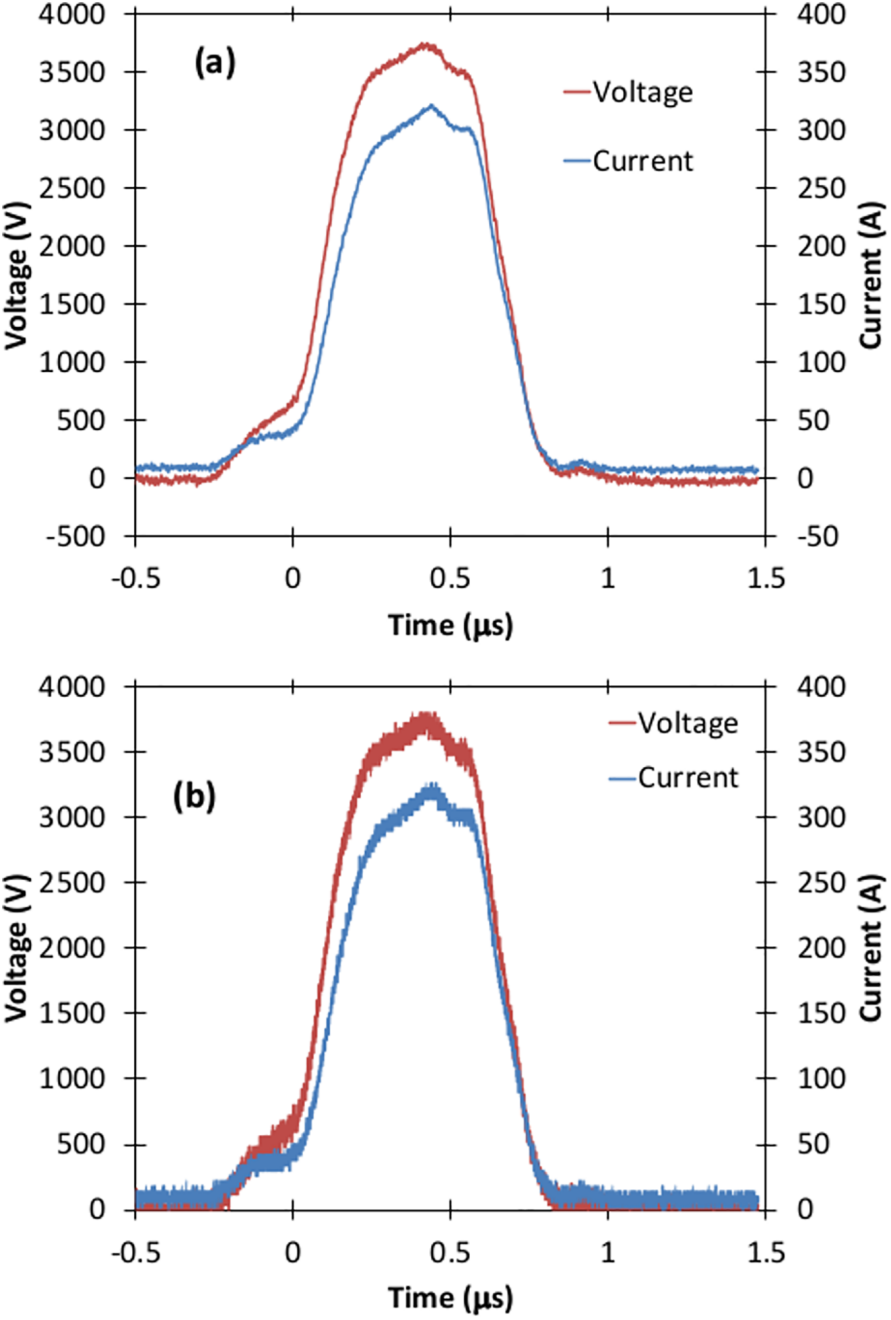
Sample voltage (blue) and current (red) traces during a 600 ns electric pulse treatment of human platelet rich plasma (PRP) samples within a 2 mm cuvette for (a) filtering using a simple moving average and (b) an unfiltered signal. The filtering does not eliminate any key features of the electric pulse.

In this subsection, we demonstrate the capability of delivering multiple pulses to PBS to explore waveform reproducibility (Fig 6), composite pulses (Fig 7), pulse compression (Fig 8), and applying electric pulses to PRP (platelet rich plasma) samples in 2 mm cuvettes (Fig 9).

Fig 6 shows voltage and current traces for five pulses applied to PBS with one minute between pulses to demonstrate the repeatability of the pulses. We chose one minute to minimize the potential contribution induced by changes in suspension conductivity due to membrane permeabilization. The resulting traces show minimal changes in both the measured voltage and current, illustrating the excellent reproducibility of electric pulses using this device.

Figs 7 and 8 show the measured voltage and current using a Tektronix DPO4104 oscilloscope with a Tektronix P6015A high voltage probe and a Pearson current probe, Model 110. Fig 7 illustrates the pulse generator’s capability of creating a composite pulse (a single pulse comprising pulses of multiple amplitudes, or a pulse of variable amplitude) with amplitudes of 5.5 kV and 1 kV and a peak current absorption of approximately 110 A (the load was a resistor). The composite pulse displayed in Fig 7 is significant because it shows that the instrument may be used for novel intracellular manipulation. A combination of submicrosecond and microsecond pulses may induce both intracellular and membrane manipulation or, at least, facilitate the selective tuning of potentially unique PEF induced biological effects. For instance, gene delivery could be facilitated if applying a microsecond pulse permeabilized the plasma membrane and the subsequent submicrosecond pulse permeabilized the nucleus. The ability to apply these pulses without delay would prevent any potential membrane resealing that might mitigate the effect.

We also experimented and collected data for solid state pulse sharpening and report initial results in Fig 8 for pulse sharpening applied to the output with a single load. In Fig 8, the horizontal scale is 200 ns per division and the vertical scale is 1 kV per division (for the blue and yellow signal) or 100 A per division (for the purple signal). The blue waveform is the voltage generated by the IGBT stages before the pulse sharpening stage, the yellow waveform represents the voltage after sharpening with a rise time of tens of nanoseconds, and the purple waveform represents the current through the load. Incorporating the pulse sharpening stage reduces the rise- and fall-times by several orders of magnitude despite the large current (peak current in excess of 350 A) absorbed by the load (whole blood in 2 mm cuvette for this test).

Fig 9A shows examples of voltage/current traces applied by our pulse generator for a 2 mm commercial cuvette containing platelet rich plasma (PRP), which was separated from whole blood from a human donor using a commercial centrifuge. We digitally filtered these traces to remove noise introduced by the measuring system by using a simple moving average filter that considers up to eleven samples with five before and five after the filtered data sample. We verified that the filter procedure did not remove features of interest in the waveforms presented here. Fig 9B shows the unfiltered signal, demonstrating that all major features remain in the filtered signal shown in Fig 9A.

The voltage and current in Fig 9 are in phase, or rise and fall simultaneously, indicating that the load (cuvette with a PRP sample) can be reasonably approximated as a resistor for establishing the load impedance of the pulse generator. The voltage rise- and fall-times are approximately 100 ns with a total pulse duration of approximately 600 ns (this pulse duration was obtained without using the pulse sharpening capabilities of this first generation prototype). The peak voltage is approximately 4 kV, which translates into an electric field of 20 kV/cm across the PRP in a 2 mm cuvette; the peak current is on the order of 300 A. The waveforms show an initial slow rise due to the delay in the communication line with the hardware and a dead-time purposely introduced between opening one set of switches and closing the second set for every stage; this dead time was introduced to avoid a short circuit during operation (to avoid having both switches per stage closed at the same time). These delays and dead-times can be optimized in the next hardware version. The prototype has an easy to use interface, enabling the user to program the desired pulse features, such as pulse amplitude, pulse duration, and number of pulses.

### Pulse generator tests in buffer solution

Following pulse generator design and construction, we initially focused on testing device flexibility by treating 2 mm cuvettes containing solutions of either PBS or low conductivity buffer.

#### Pulse generator validation for a high conductivity buffer (PBS)

**Fig 10:**
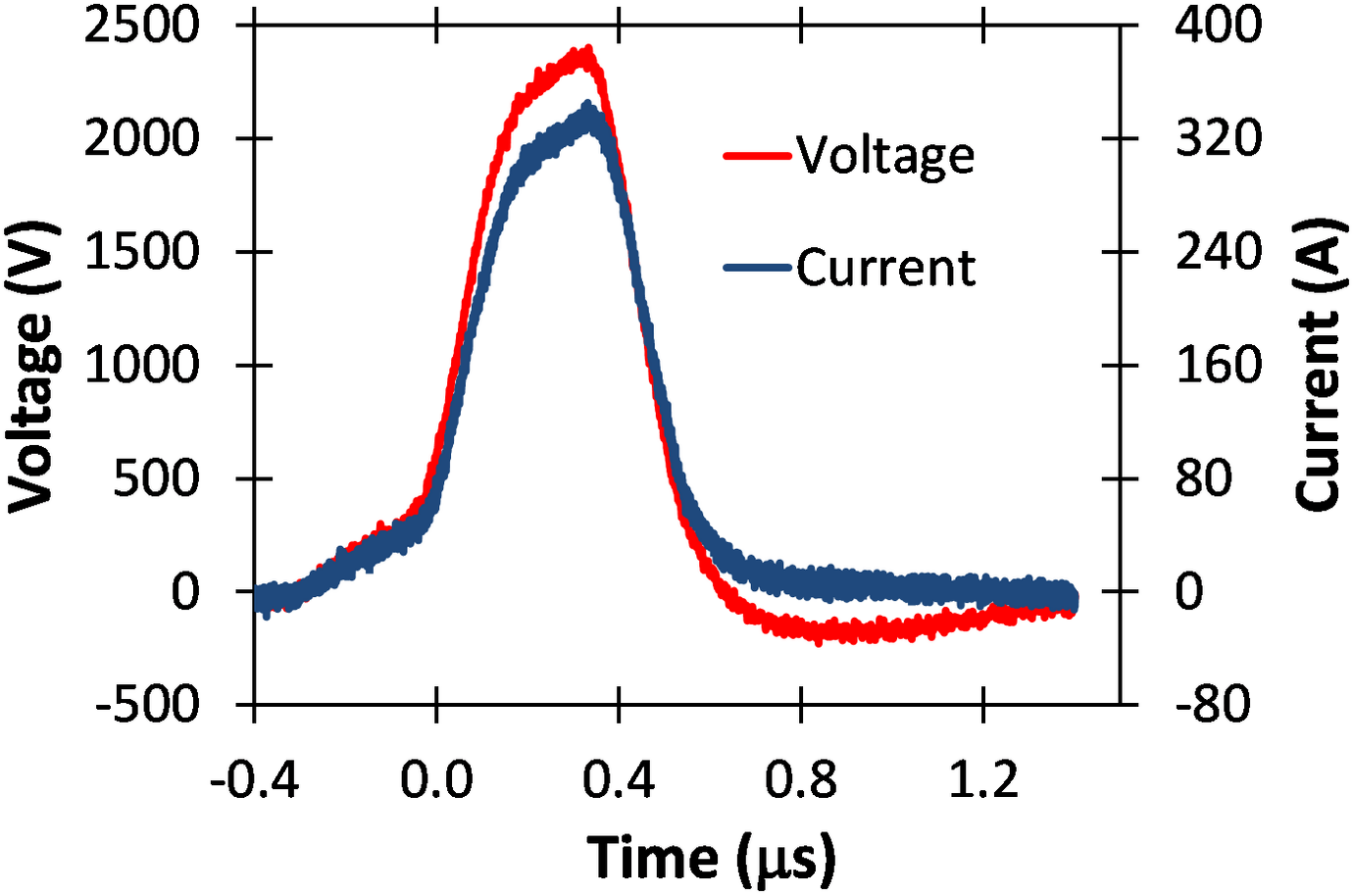
Voltage and current waveforms for a 400 ns pulse of approximately 2500 V applied to a 2 mm cuvette containing PBS.

**Fig 11:**
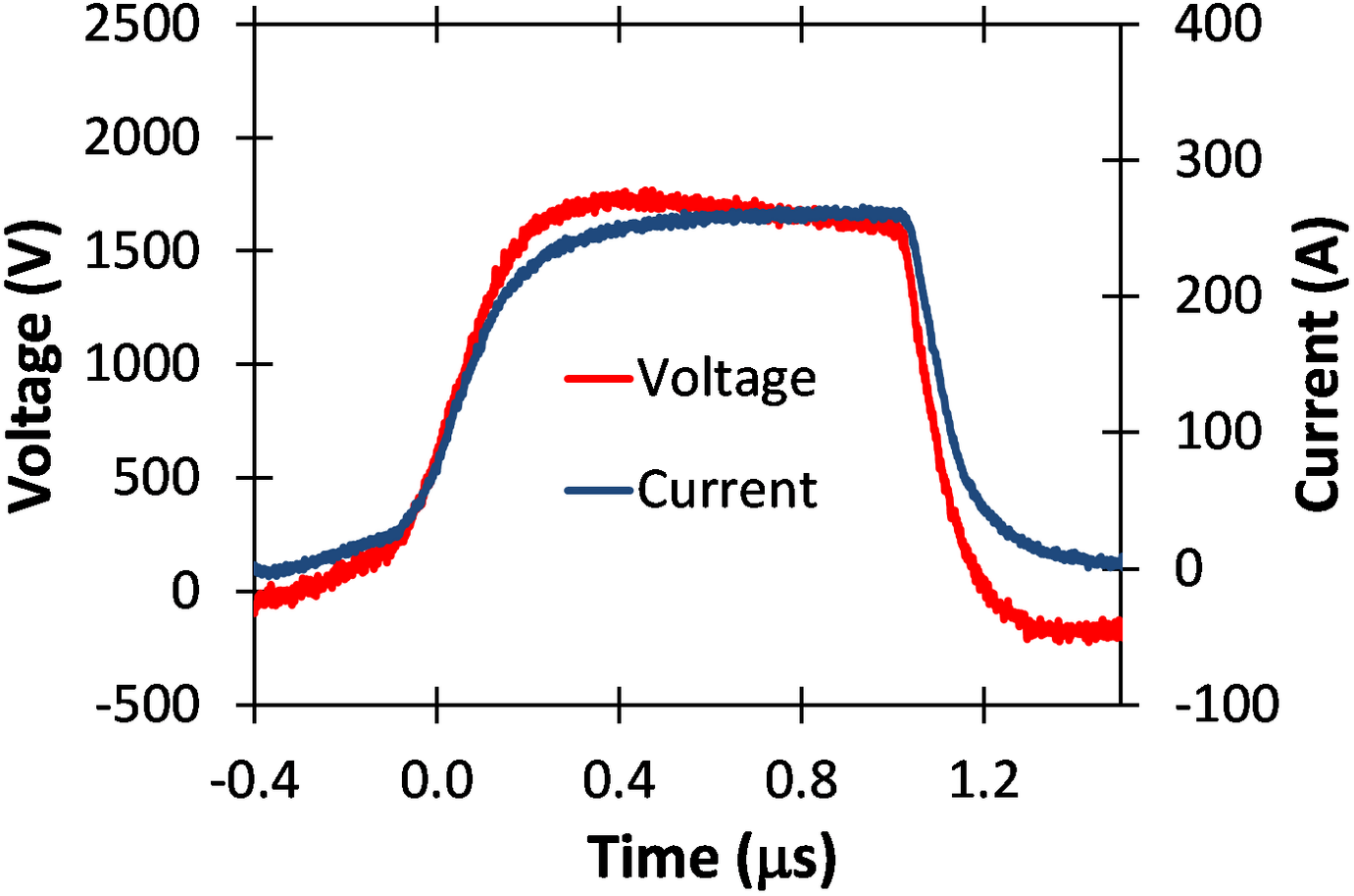
Voltage and current waveforms for a 1 μs pulse of 1800 V applied to a 2 mm cuvette containing PBS.

**Fig 12:**
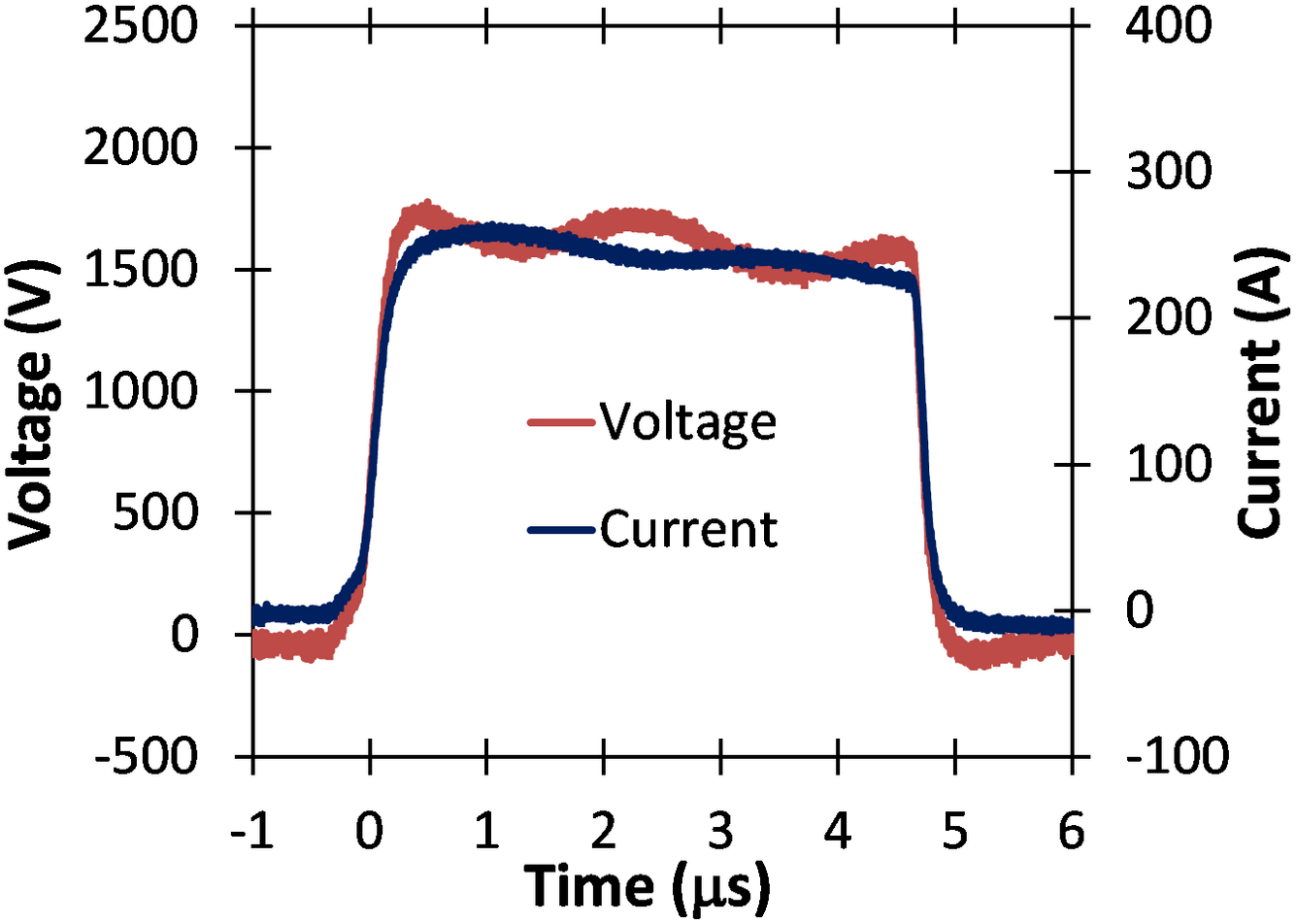
Voltage and current waveforms for a 5 μs pulse of about 1800 V applied to a 2 mm cuvette containing PBS.

**Fig 13:**
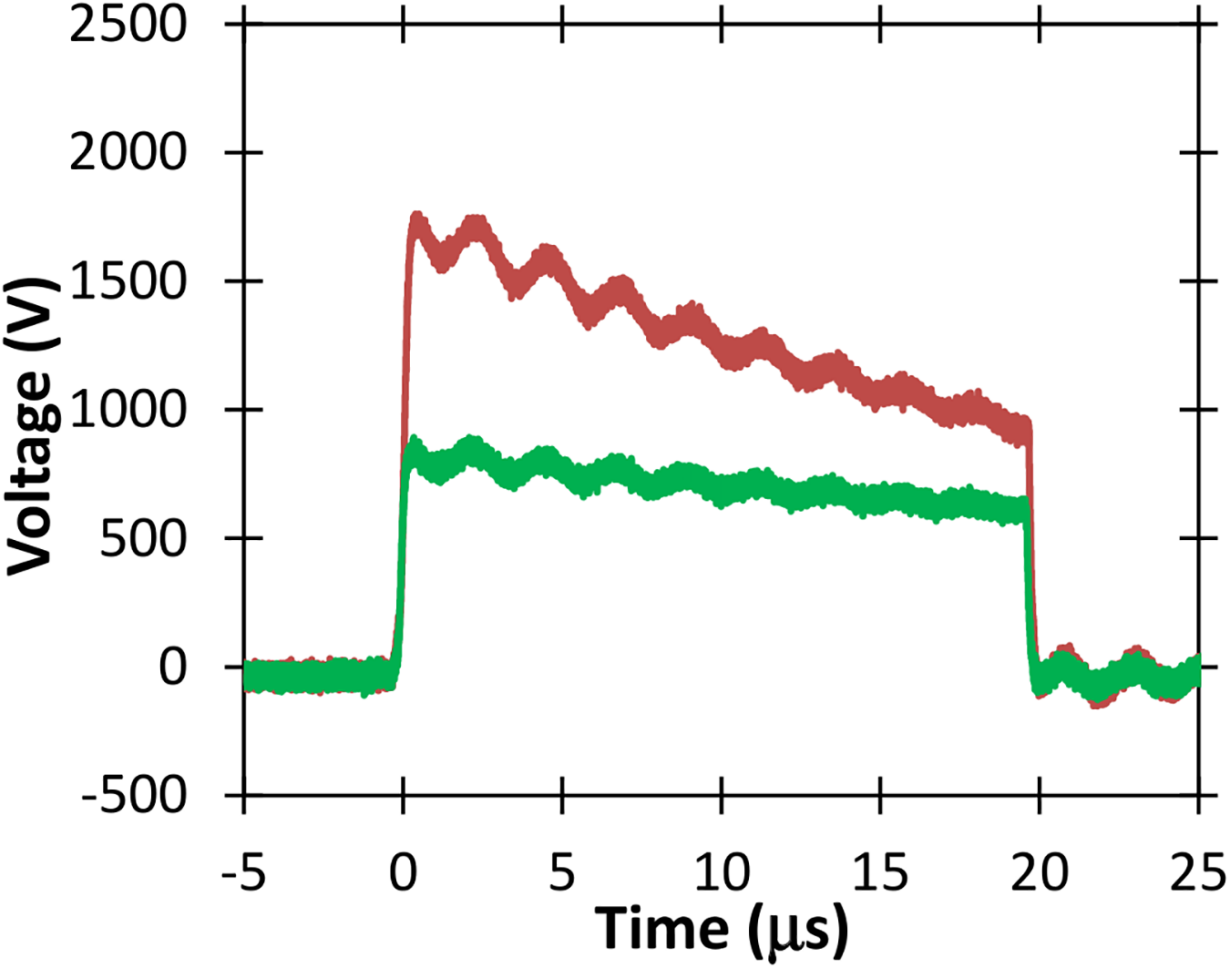
Voltage waveforms for a 20 μs pulse of about 1800 V applied to a 2 mm cuvette containing PBS.

Figs 10, 11, 12, and 13 show various voltage waveforms delivered by the GE Global Research pulse generator for various voltages and pulse widths that the user can readily change using the custom, laptop based control software. PBS has a higher conductivity than typical electroporation buffers.

**Fig 14:**
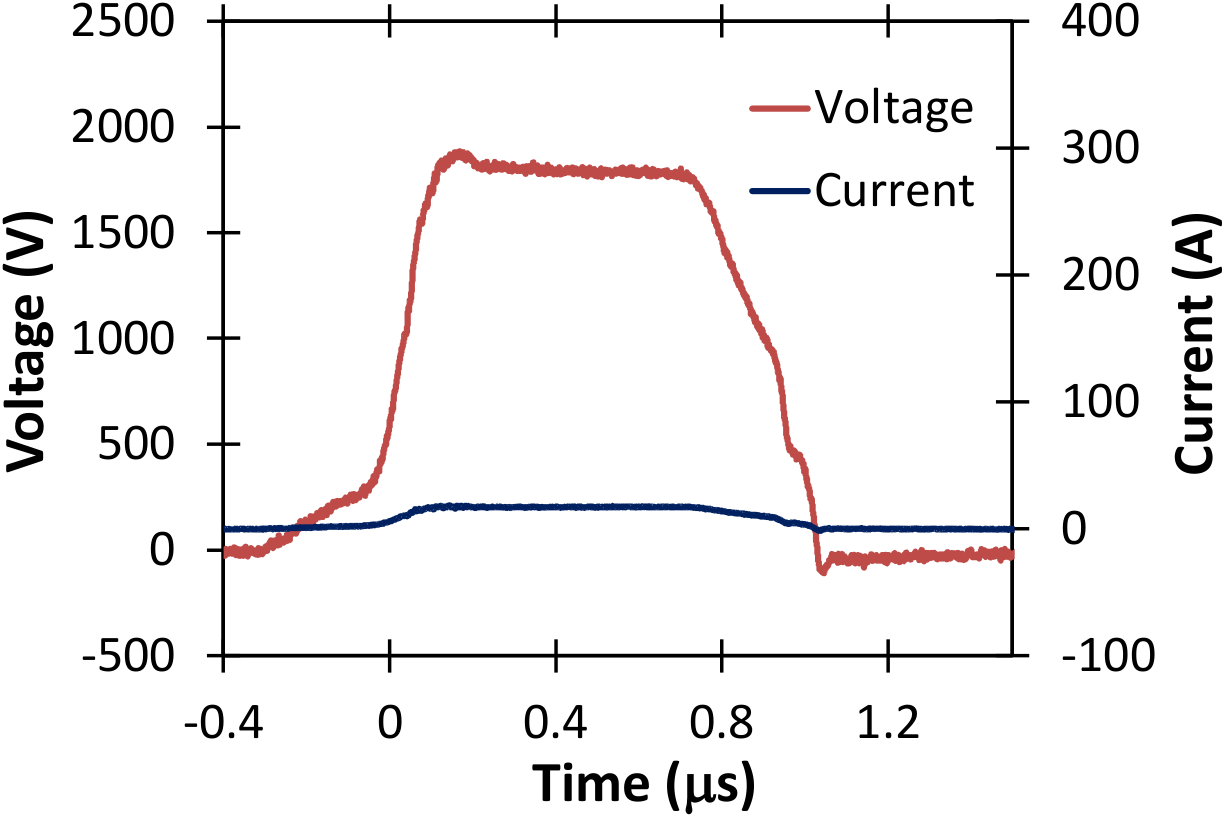
Voltage and current measurements during the application of a 1 μs electric pulse to a 2 mm cuvette with low conductivity buffer.

The oscillations in Figs 12 and 13 arise because of parasitic inductance (~80 nH) associated with the leads connecting the load to the pulse generator, and the filtering capacitors (the blue components shown in Fig 5B) with the characteristic impedance ~0.2 Ω when all stages are connected. The oscillations are larger for higher conductivity samples, as shown in Fig 14. The oscillations are also not apparent for pulses much shorter than the resonant period, which can be calculated as approximately 2.2 μs based on Figs 12 and 13. Thus, one would not anticipate any oscillations for pulses shorter than approximately 1 μs.

#### Pulse generator validation for a low conductivity buffer

To demonstrate the impact of buffer conductivity on the GE Global Research pulse generator, we also treated a low conductivity buffer with electric pulses. We used a low conductivity phosphate buffer consisting of 10 mM KH_2_PO_4_, 1 mM MgCl_2_, and 250 mM sucrose in sterile water while fixing the pH at 7.4 [51]. Fig 14 shows the resulting voltage and current traces following exposure of a 2 mm cuvette to a 1 μs pulse with a peak voltage of approximately 1.8 kV. The current for the low conductivity buffer is approximately a factor of ten lower than for the PBS shown in Fig 11 for the same pulse duration and approximately same peak voltage. This indicates that the conductivity of PBS is a factor of ten higher than that of the low conductivity buffer.

**Fig 15:**
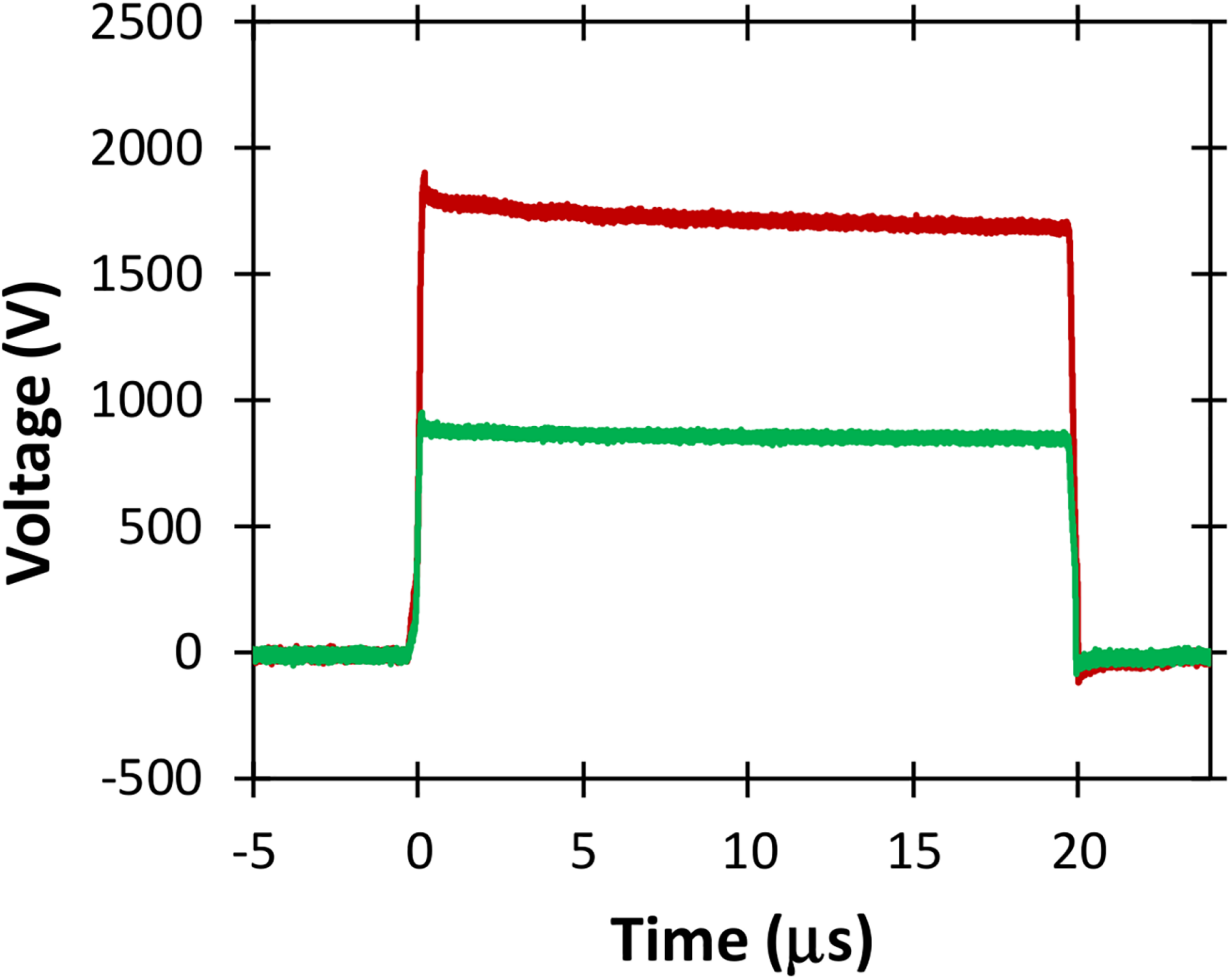
Voltage waveforms for 20 μs pulses of various voltages applied to a 2 mm cuvette containing a low conductivity buffer. Note the relative absence of droop compared to the higher conductivity PBS sample of Fig 11.

Fig 15 shows voltage measurements for two different amplitudes of 20 μs electric pulses without the droop demonstrated for similar amplitude and duration pulses applied to the higher conductivity PBS sample (c.f. Fig 13). The absence of any droop here, as illustrated in Fig 15, indicates that the pulse generator can be operated efficiently for the low conductivity buffer.

These results demonstrate the flexibility of this device to deal with large changes in buffer conductivity at various pulse widths and voltages, since standard pulse generators require matching resistors to deal with such large changes in impedance [21]. This flexibility is particularly valuable for in vitro PEF experiments since adjusting extracellular conductivity can change the electroporation threshold or, in other words, the sensitivity of the cells to PEF application [52–54].

#### Electric pulse application for platelet rich plasma activation

We now turn to a biomedical application of PEFs using the flexible pulse generator described in detail above. Recent studies [8, 45] have explored the ability of nanosecond PEFs (nsPEFs) to activate platelets ex vivo without bovine thrombin. While bovine thrombin is the typical clinical platelet activator utilized in wound healing workflows [55–56], it may trigger potentially significant side effects [56]. Using electric stimulation for ex vivo platelet activation may provide the first non-biochemical means to release growth factors from platelets, to initiate and drive the healing cascade upon application to a wound. Electric simulation of platelets can be inexpensive and easy to use, with no known side effects [56]. A proposed mechanism for platelet activation via electric stimulation states that nsPEFs may induce Ca^2+^ release from intracellular structures or facilitate Ca^2+^ transport from the extracellular fluid to the cytoplasm through nanopores to activate platelets [8]. Such ion motion will depend upon pulse duration and intensity, so the flexible pulse generator described here will enable a detailed study of the pulse parameter space for elucidating the mechanisms involved in PEF induced platelet activation. We report here a sample case for 400 ns pulses and have recently extended these studies to 5 μs pulses using this pulse generator [56].

#### Experimental measurements of growth factors

**Fig 16:**
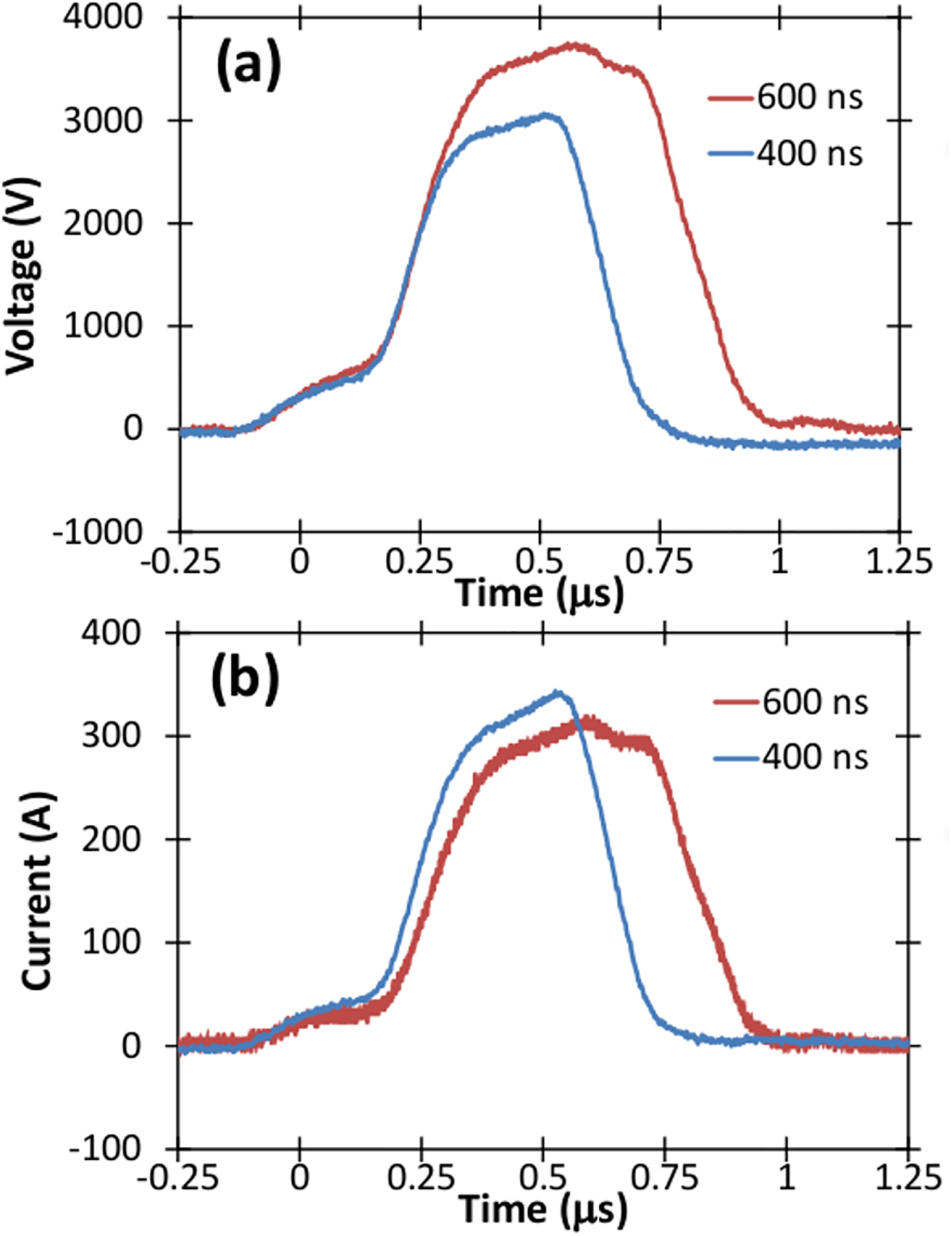
(a) Voltage and (b) Current traces for 400 ns and 600 ns pulses tested for platelet activation.

Our previous platelet activation experiments used pulses on the order of hundreds of nanoseconds [45]. While this previous work used pulses of approximately 600 ns duration [45], we utilize 400 ns pulses here. The ability to change the pulse width from 400 to 600 ns on a high conductivity load, such as PRP, further demonstrates our instrument’s flexibility. Fig 16 compares the voltage and current signals from 600 ns pulses [45] and 400 ns pulses used here.

**Fig 17:**
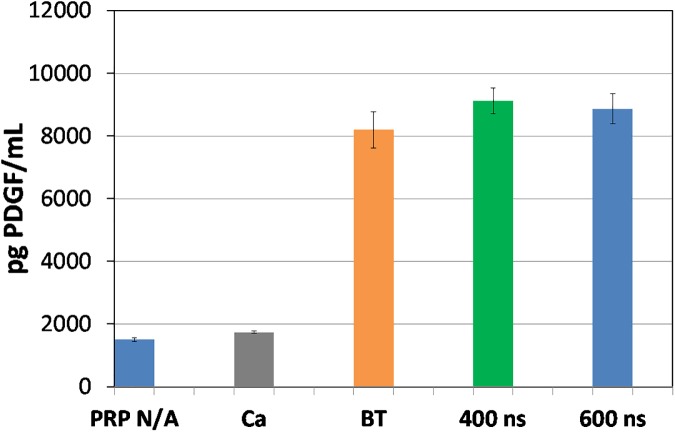
PDGF-aa for non-activated PRP (PRP N/A), PRP treated with Ca^2+^ alone (Ca), PRP activated with bovine thrombin (BT), and pulsed electric fields (PEFs) of 400 ns and 600 ns duration. Reported data comes from the average of three measurements with the error bars representing standard deviation.

**Fig 18:**
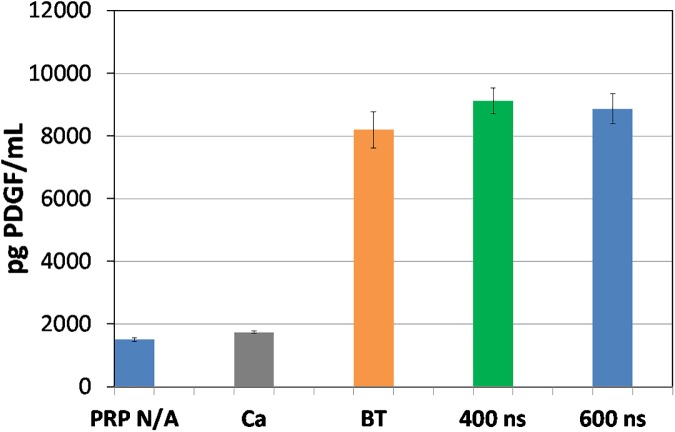
EGF for PRP (not activated), PRP treated with Ca^2+^ alone (Ca), PRP activated with bovine thrombin (BT), and pulsed electric fields (PEFs) of 400 ns and 600 ns duration. Reported data comes from the average of three measurements with the error bars representing standard deviation.

Figs 17 and 18 present PDGF and EGF release results as the average of three separate measurements with error bars determined by standard deviation. These results confirm our previous published findings [45] that PDGF is released at the same level as thrombin, while EGF is released at much higher level compared to thrombin. We have also used this pulse generator to apply 5 μs pulses to activate platelets with similar levels of PDGF and EGF [56]. Thus, these results indicate that both submicrosecond and microsecond duration pulses can activate platelets. At present, these results may suggest primarily plasma membrane level effects since the 400 ns and 600 ns pulses induce nanopores that may facilitate Ca^2+^ transport into the cell to activate platelets and the 5 μs pulses almost certainly can induce pore formation. However, electroporation pulses may also induce intracellular effects in addition to membrane level ones [14], so future studies exploring the impact of these pulses on both the membrane and intracellular calcium stores, such as the endoplasmic reticulum, are required to more clearly understand the mechanisms involved. Additional studies will also elucidate the impact of pulse parameters on growth factor release, the subsequent impact of these growth factors on wound healing in pre-clinical and clinical testing, and the possibility of tuning activation as a function of electric pulse parameters.

## Discussion

We have demonstrated a modified, compact Marx architecture capable of generating pulses from hundreds of nanoseconds (which can be modified to tens of nanoseconds) to tens of microseconds in duration, with unprecedented flexibility in terms of pulse shape for bioelectrics research. While we have demonstrated pulse durations of 400 ns here, we can use the pulse sharpening hardware whose impact is demonstrated in Fig 8 to further shorten the pulse duration. Specifically, Fig 8 shows that the pulse sharpening hardware can shorten the pulse duration by approximately 300 ns at the rise (beginning) of the pulse. Using pulse sharpening at both the beginning and end of the pulse can reduce the pulse width from the present 400 ns duration (c.f. Fig 16) to tens of nanoseconds if future applications demand it (the current application of platelet activation does not require pulses this short).

This pulse generator can also create novel pulse waveforms, such as superimposing nanosecond and microsecond pulses into a single waveform (composite pulses, or pulse of variable amplitude). Such a novel shaped pulse could simultaneously target the plasma membrane and intracellular organelles to tune specific biological effects, such as electroporation or permeabilization of intracellular organelles. Future simulation studies could provide insight into potential implications on membrane pore dynamics and membrane voltage development due to these novel waveforms.

Moreover, the ability to treat suspensions of multiple conductivities is particularly important for in vitro and ex vivo applications. Typical electroporators are designed for low conductivity buffers, which tend to be the easiest and least expensive to design for biomedical applications. In fact, many commercial devices use special proprietary buffer solutions to optimize molecular delivery to cells. While effective, this does not provide the user with flexibility for treating suspensions of higher conductivity, such as the PRP samples tested above or even treating cells in growth media when it may be advantageous. A tunable pulse generator as described here will enable the user to treat a wide spectrum of samples from standard electroporation samples to highly conductive ex vivo, and perhaps even in vivo, samples. Additionally, it would easily allow manipulation of extracellular conductivity for optimizing desired effects, such as electroporation [52–54].

We have also demonstrated that this pulse generator can successfully activate platelets for 400 ns and 600 ns pulses and induce similar PDGF release levels as bovine thrombin and higher levels of EGF release than bovine thrombin. These results motivate future animal studies to assess the capability of platelets activated by nsPEFs to induce similar or improved wound healing compared to bovine thrombin activated PRP.

In summary, this pulsed power topology [57] will provide an ultra-flexible device for examining PEF interactions with cells for a wide variety of pulse durations, pulse shapes, and solution conductivities. Upon system optimization, it could be developed into a commercial device that could be used in the clinic for treating ex vivo autologous samples, such as PRP [58]. The clinical workflow will include similar steps for PRP separation from whole blood by centrifugation. Activation by electric stimulation at the bedside would be accomplished by pressing a button on the pulse generator rather than by using bovine thrombin. This will provide the clinician with the first instrument for inducing PRP growth factor release from platelets using a platelet activation technique that is less expensive and easier to use than the conventional bovine thrombin technique, with a workflow that does not require an animal derived activator.

## Supporting information

S1 Dataset Dataset for Fig 1. (XLSX)

S2 Dataset Dataset for Fig 2. (XLSX)

S3 Dataset Dataset for Fig 6. (XLSX)

S4 Dataset Dataset for Fig 9. (XLSX)

S5 Dataset Dataset for Fig 10. (XLSX)

S6 Dataset Dataset for Fig 11. (XLSX)

S7 Dataset Dataset for Fig 12. (XLSX)

S8 Dataset Dataset for Fig 14. (XLSX)

S9 Dataset Dataset for Fig 16. (XLSX)

S10 Dataset Dataset for Fig 17. (XLSX)

S11 Dataset Dataset for Fig 18. (XLSX)
